# Rational Design of Lewis Base Electron Transport Materials for Improved Interface Property in Inverted Perovskite Solar Cells: A Theoretical Investigation

**DOI:** 10.3390/nano13091560

**Published:** 2023-05-05

**Authors:** Xueqin Ran, Jixuan Yang, Mohamad Akbar Ali, Lei Yang, Yonghua Chen

**Affiliations:** 1 Key Laboratory of Flexible Electronics (KLOFE) & Institution of Advanced Materials (IAM), School of Flexible Electronics (Future Technologies), Nanjing Tech University (Nanjing Tech), Nanjing 211816, China202161122151@njtech.edu.cn (J.Y.);; 2 Advanced Materials Chemistry Center (AMCC), Khalifa University of Science and Technology, Abu Dhabi P.O. Box 127788, United Arab Emirates; 3 Department of Chemistry, College of Art and Science, Khalifa University of Science and Technology, Abu Dhabi P.O. Box 127788, United Arab Emirates; 4 Centre for Molecular Systems and Organic Devices (CMSOD), Key Laboratory for Organic Electronics and Information Displays & Jiangsu Key Laboratory for Biosensors, Institute of Advanced Materials (IAM), Jiangsu National Synergetic Innovation Center for Advanced Materials (SICAM), Nanjing University of Posts & Telecommunications, 9 Wenyuan Road, Nanjing 210023, China

**Keywords:** electron transport material, theoretical design, Lewis base, heteroatoms, perovskite/ETM interface

## Abstract

Electron transport materials (ETMs) play a vital role in electron extraction and transport at the perovskite/ETM interface of inverted perovskite solar cells (PSCs) and are useful in power conversion efficiency (PCE), which is limited by interface carrier recombination. However, strategies for passivating undercoordinated Pb^2+^ at the perovskite/ETM interface employing ETMs remain a challenge. In this work, a variety of heteroatoms were used to strengthen the Lewis base property of new ETMs (asymmetrical perylene-diimide), aimed at deactivating non-bonded Pb^2+^ at the perovskite surface through Lewis acid-base coordination. Quantum chemical analysis revealed that novel ETMs have matched the energy level of perovskite, which enables electron extraction at the perovskite/ETM interface. The results also suggest that the large electron mobility (0.57~5.94 cm^2^ V^−1^ s^−1^) of designed ETMs shows excellent electron transporting ability. More importantly, reinforced interaction between new ETMs and Pb^2+^ was found, which is facilitating to passivation of the defects induced by unsaturated Pb^2+^ at the perovskite/ETM interface. Furthermore, it is found that MA (CH_3_NH_3_^+^), Pb, and I_Pb_ (iodine substituted on the Pb site) defects at the perovskite/ETM interface could be effectively deactivated by the new ETMs. This study provides a useful strategy to design ETMs for improving the interface property in PSCs.

## 1. Introduction

In recent years, many researchers have been attracted to inverted lead halide perovskite solar cells (PSCs) due to their exceptional properties, including high absorption coefficient, long charge diffusion length, and large bandgap range [1,2,3,4]. PSC efficiency has been significantly improved due to the rapid development of compositional engineering, additive engineering, and interface engineering, and is now comparable to that of the best single-crystalline silicon solar cells [5,6]. However, due to PSCs’ lower efficiency than the theoretical efficiency limit and their poor stability when influenced by moisture, oxygen, light, and heat, researchers are motivated to seek an alternative solution to resolve these problems [7,8,9,10,11]. In addition to compositional engineering and additive engineering, developing high performance electron transport materials (ETMs) was suggested as an effective solution that could improve the interface properties as well as the efficiency and stability of PSCs [12,13,14,15]. This is realized by the decreasing charge recombination from the effective electron extraction at the perovskite/ETM interface and fluent electron transportation in the whole device [16]. Therefore, our group was motivated to understand an effective way to tailor the geometries of ETMs, which can improve the efficiency and stability of PSCs. Compared with inorganic ETMs, such as TiO_2_, traditional organic ETMs, such as fullerene and its derivatives [6,6]-phenyl-C_61_-butyric acid methyl ester (PCBM), have many specific advantages, for example: good electron extraction ability, simple fabrication, good compatibility with flexible substrates, low solution-processing temperature, etc. [17]. Unfortunately, due to the high price of PCBM and its sensitivity to reaction with oxygen, many research groups are interested in seeking alternatives to traditional organic ETMs with high performance [18,19].

Recently, various small organic molecules have been synthesized and used as ETMs for PSCs, including pyrazine-azaacene, naphthalene-diimide, perylene-diimide, and their derivatives [20,21,22,23]. Their intrinsic advantages, such as good repeatability and high purity and mobility, have led to their increased use in PSCs. For example, Chu’s group reported tetrachloro-substituted perylene-diimides as new efficient ETMs. They demonstrated good charge-transport properties and improved the stability of PSCs [24]. Sugura et al., and Zhu et al., proposed a novel ETM, i.e., hexaazatriphenylene derivatives with high performance [25,26]. These ETMs with nanostructures may exhibit a wide range of electrical and optical properties that depend sensitively on both their shapes and sizes, and thus are likely to provide a new method for modifying the optical and electronic properties of organic functional materials [20,21,22,23,24,25,26]. In addition, the possibility of using nanostructures in the performance of solar cells was demonstrated and summarized in previous works [27,28,29,30].

Among a variety of smaller ETMs, PDIs (perylene-diimide derivatives) have attracted much attention due to their use in commercially available raw materials, high electron mobility, and stability [31,32,33,34]. Compared with symmetrically substituted structures, asymmetrical PDIs with multifunctional groups show strengthened π-π stacking and electrostatic and H-bonding interactions, which is beneficial for achieving high performance PSCs [35].

**Figure 1:**
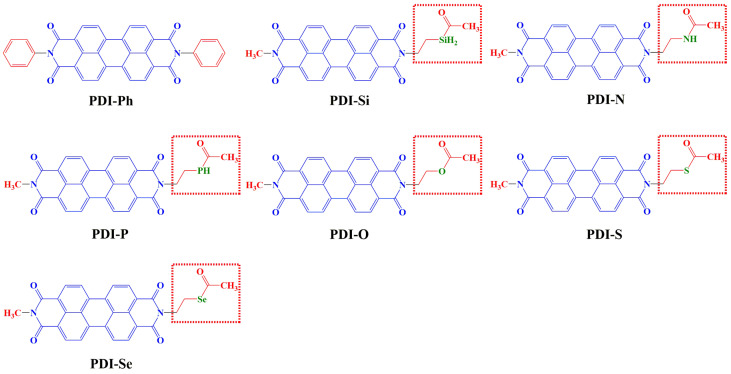
Framework of the available ETM (PDI-Ph) and designed ETMs (PDI-Si, PDI-N, PDI-P, PDI-O, PDI-S, and PDI-Se) (own elaboration).

Moreover, a variety of solvents and additives with a heteroatom donor, i.e., nitrogen, oxygen, and sulfur, were widely reported as Lewis bases to passivate the Lewis acid Pb^2+^ in perovskite photovoltaic devices [36]. It was found that these polar atoms could stabilize unsaturated Pb^2+^ and improve the performance of perovskite [2,4]. Based on this concept, silicon, nitrogen, phosphorus, oxygen, sulfur, and selenium atoms were introduced in asymmetrical perylene-diimides, as shown in Figure 1, to design a new class of ETMs, i.e., PDI-Si, PDI-N, PDI-P, PDI-O, PDI-S, and PDI-Se, with improved Lewis base properties. The designed PDIs are shown in Figure 1. The related performance of the novel ETMs in nano-size was investigated theoretically and compared with a known ETM PDI-Ph (Figure 1). The aim of the present work is to enhance the electron extraction/transport at the perovskite/ETM interface and decrease the defects derived from uncoordinated Pb^2+^, which can be helpful for achieving high-performance perovskite photovoltaic devices. The different heteroatoms in the structure adjust the geometry, stability, solubility, frontier molecular orbital property, UV-visible absorption spectrum, electron transporting, π-π stacking, electrostatic and non-covalent interaction of ETMs, and perovskite/ETM interface interaction.

## 2. Computational Methods

Six asymmetrical perylene-diimides (As-PDIs) with different Lewis base heteroatoms (PDI-Si, PDI-N, PDI-P, PDI-O, PDI-S, and PDI-Se) were designed and compared with symmetrical perylene-diimide PDI-Ph. The chemical nomenclatures of these PDIs are provided in Supplementary Materials. In As-PDIs, heteroatom-substituted ketone is connected to one of the active nitrogen atoms in the perylene-diimide backbone while methyl linked with another nitrogen. The phenyl substituted on each end-nitrogen atom of perylene diimide in PDI-Ph. The ground state geometries of the PDI derivatives were optimized by employing hybrid density functional theory (DFT). Then, vibrational frequencies of these molecules were calculated using DFT methods to estimate the zero-point corrections. The optimized geometrical parameters and normal modes of frequencies were tabulated in Table S2. The positive vibrational frequencies indicate that all optimized structures have minimal energies in potential energy surface (see Table S2). On the stable geometries of PDI derivatives, frontier molecular orbitals and relevant electronic properties were investigated utilizing DFT methods. It is known that suitable HOMO (highest occupied molecular orbital) and LUMO (lowest unoccupied molecular orbital) energies are preliminary requirements for ETMs and are the key factors for open-circuit voltage (*V*oc) in PSCs. In order to obtain more accurate HOMO and LUMO energies, which depend on DFT methods, a few of the most popular functionals, i.e., B3LYP, PBE1PBE, MPW1B95, PBE33, PBE38, and MPWB1K, were compared together with the 6-31+G(d,p) basis set (see Table S3). It was found that B3LYP provided the energy that was most similar to HOMO and LUMO energies of the available experimental data; this was also consistent with our previous work [37]. Therefore, B3LYP/6-31+G(d,p) were employed to calculate the geometries and electronic properties for all the PDIs. The ionization potentials and electron affinities with adiabatic excitations were obtained on the basis of geometries in neutral, positive, and negative status. The solvation free energy (∆G^0^_solv_) was calculated as the energy difference of materials in gas and solution phases with a polarizable continuum model (PCM) in the toluene solvent [38,39,40]. The UV-visible absorption spectrum and maximum wavelength (λ_max_) were obtained by employing time-dependent density functional theory (TD-DFT). The calculations in this part, including ground state geometries, vibrational frequencies, HOMO, LUMO, ionization potentials, electron affinities, ∆G^0^_solv_, and λ_max_ for the PDIs, were carried out using the Gaussian 09 suite of programs (a general computational chemistry software) [41].

In organic molecular materials, charge carriers generally localize on a single molecule and randomly move to neighboring molecules through a hopping mechanism, for which the charge hopping rate could be predicted successfully by the classical Marcus Hush formula [42], as provided in Equation (1):

(1) $$k={\left(\frac{\pi }{\lambda k_{B}T}\right)}^{\frac{1}{2}}\frac{V^{2}}{\hbar }\exp\left(-\frac{\lambda }{4K_{B}T}\right)$$

where k denotes electron transfer rate, λ is electron reorganization energy due to geometric relaxation in the process of electron transfer, k_B_ is the Boltzmann’s constant, T is temperature, *ħ* ($\frac{h}{2\pi })$, is reduced Planck’s constant, and V is electron transfer integral between two species that are dominated mostly by orbital overlap, respectively.

Compared to inner reorganization, external reorganization contributes in a very small manner; therefore, only the inner reorganization was calculated. The inner reorganization was calculated on the adiabatic potential energy surface (APES) [43,44,45] for PDIs.

The electron transfer integral (V) was calculated by the formula provided in Equation (2) [46,47]:

(2) $$V=\langle \varphi_{i}^{HOMO/LUMO}\left|F\right|\varphi_{f}^{HOMO/LUMO}\rangle$$

where φ^HOMO/LUMO^ represents the HOMO or the LUMO of the isolated molecule in the dimer and F is the Fock operator of the dimer.

To understand the transport capacity of proposed PDIs, the carrier mobility was calculated by the Einstein relation, as provided in Equation (3) [48]:

(3) $$\mu =\frac{1}{2n}\frac{e}{k_{B}T}\sum_{i}r_{i}^{2}k_{i}P_{i}$$

where *n* is the dimensionality of crystal, *r_i_* is the distance between neighboring molecules in crystal, *k_i_* is charge hopping rate, and *P_i_* is fraction of charge hopping rate as expressed in Equation (4):

(4) $$P_{i}=\frac{k_{i}}{\sum_{i}k_{i}}$$

For the purpose of obtaining the crystal structures of PDIs, a single molecular structure was optimized in BIOVIA Materials Studio Dmol3 model (a program utilizing DFT to simulate chemical processes and predict properties of materials both rapidly and accurately), using GGA-PBE (Perdew–Burke-Ernzerhof) functional with an accuracy of 10^−5^ [49]. The crystal structures of these molecules were then predicted in BIOVIA Materials Studio Polymorph model (a program that can be used to predict potential polymorphs of a given compound directly from the molecular structure) under the Dreiding force field [49]. The most probable space groups, i.e., *P*1, *P*2_1_*/C*, *P*2_1_2_1_2_1_, *C*2*/C*, and *P*2_1_, were restricted during crystal prediction. In order to estimate the interaction between proposed PDIs and the perovskite system, the interaction energies of PDIs and the group/atom (MA, Pb, and I) on the surface of perovskite were fully investigated using the Dmol3 program. Once these interactions were obtained, the geometry of ETM on perovskite surface was investigated using GGA-PBE functional in the BIOVIA Materials Studio CASTEP model (an ab initio quantum mechanical program employing DFT to simulate the properties of solids, interfaces, and surfaces for a wide range of materials classes such as ceramics, semiconductors, and metals) [49]. The density of states (DOS) and the defect formation energy of MA, Pb, and I_Pb_ for the PDIs adsorbed on the perovskite surface were calculated. For CASTEP calculation, the vacuum region was set to 25 Å, the quality of k-points was set to fine, and the convergence tolerance of energy was set to 2.0 × 10^−5^ eV per atom. All the geometries were relaxed until a residual force less than 0.05 eV Å^−1^ per atom was obtained. The calculations in this part were performed by Material Studio 8.0 software package [49].

## 3. Results and Discussion

### 3.1. Geometric and Electronic Structures

**Figure 2:**
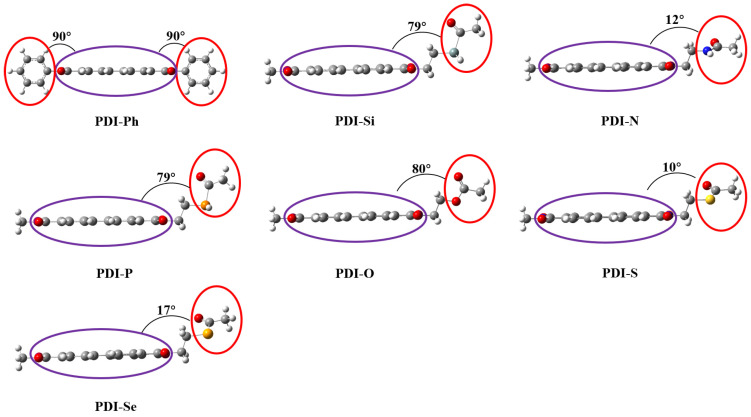
The optimized geometries of investigated PDIs. The dihedral angles are shown on each structure. The optimized parameters of all PDIs are provided in the Supplementary Materials Table S2.

The optimized ground state geometries for all PDIs are shown in Figure 2. The variation of substituted functional groups on PDI can regulate the molecular and crystal geometries. The optimized geometries indicate that the relevant bond length and bond angle are similar in all the PDIs molecules. It was found that the calculated dihedral angle between the phenyl ring and the PDI skeleton in PDI-Ph was 90 degrees. In As-PDIs, the dihedral angles between PDI skeleton and functional groups were between ~12 and ~80 degrees. The large difference in dihedral angles of As-PDIs could be attributed to the variation of Lewis base heteroatoms. Chemical modification in these PDIs can cause the transformation of π-π stacking and H-bonding, which have a great influence on electron transfer performance.

**Figure 3:**
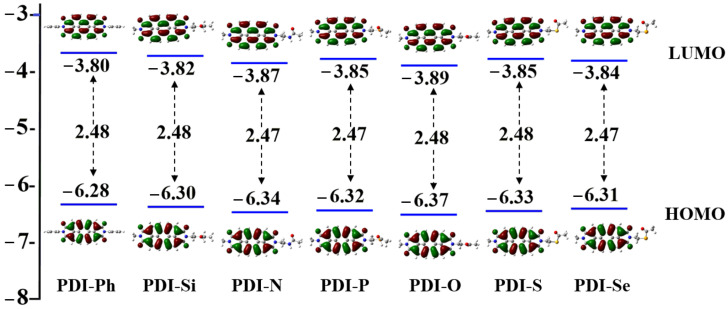
Spatial distribution and energies of HOMO and LUMO, as well as the HOMO-LUMO energy gaps for studied molecules.

The properties of frontier molecular orbital (FMO), including the HOMO, LUMO, and LUMO-HOMO gap, were calculated for the PDIs, as shown in Figure 3. It can be seen that asymmetrical geometries and various heteroatoms have little effect on the distribution of HOMO and LUMO, which mainly originate from the contribution of the PDI backbone. In PDI-Ph and As-PDIs, the HOMO and LUMO are fully delocalized on the PDI core, respectively, suggesting strong π-π intermolecular interactions, which increase the possibilities of π-π overlap between neighboring moieties and improve carrier transfer. As suggested in an earlier study, the experimentally measured HOMO (−5.98 eV) and LUMO (−3.92 eV) energies for PDI-Ph are in the range of energy levels for ideal ETMs (HOMOs are below −5.40 eV, LUMOs are between −4.20 and −3.90 eV) in MAPbI_3_ PSCs [50]. There is a small difference between the calculated HOMO (−6.28 eV) and LUMO (−3.80 eV) energies of PDI-Ph (0.30 eV for HOMO and 0.12 eV for LUMO) with experimentally measured data. The values for designed PDIs are slightly smaller (with a deviation of 0.02~0.09 eV) than PDI-Ph. The results indicate that designed molecules have well-matched energy levels with MAPbI_3_, which may allow the electrons to transfer frequently from perovskite to ETMs; on the other hand, holes may be prevented from perovskite to ETMs [51]. As a result, hole-electron recombination could be largely decreased, which is beneficial for the improvement of PSC performance. In addition, it is worth noting that the LUMO-HOMO energy gaps for PDI-Si (2.48 eV) PDI-N (2.47 eV), PDI-P (2.47 eV), PDI-O (2.48 eV), PDI-S (2.48 eV), and PDI-Se (2.47 eV) are adjacent to the value of PDI-Ph (2.48 eV), indicating similar abilities to prohibit hole-electron recombination in asymmetrical and symmetrical ETMs. In addition, the smaller LUMO energies in As-PDIs compared to PDI-Ph explain the better electron extraction ability and environmental stability, ascribed to the introduction of a Lewis base heteroatom [52].

To obtain further insight on the influence of heteroatoms on the property of designed PDIs, the UV visible spectrum was calculated on the basis of the lowest singlet-singlet vertical transition, as shown in Supplementary Materials (Figure S1). The maximum wavelengths (λ_max_), microscopic information about the oscillator strengths, excitation energy, electronic transitions, and main configuration for these molecules are listed in Supplementary Materials (Table S4). Interestingly, all these PDI molecules show the electronic π → π* from S_0_ to S_1_ state, with the main contribution derived from HOMO to LUMO. The calculated λ_max_ for PDI-Ph (527.02 nm) is in good agreement with the experimentally measured value (550 nm) [34], which provides more confidence in our theoretical methods. The λ_max_ in absorption spectra is blue-shifted a little in designed PDIs (525.84~526.36 nm) compared to PDI-Ph (527.02 nm), ascribing to the introduction of Lewis base heteroatom in As-PDIs. Similar values were found in the λ_max_ of As-PDIs, with a deviation smaller than 0.52 nm, suggesting the variation of the heteroatom in designed PDIs has little influence on the absorption maximum wavelength.

### 3.2. Stability and Solubility

As potential ETMs formed on top of the perovskite layer in inverted PSCs, new PDIs may protect perovskite materials from the destruction of O_2_ and H_2_O, thus leading to better PSCs performance. To evaluate the air stability of anions and neutral molecules against O_2_ and H_2_O, adiabatic electron affinity (EA_a_) is calculated. The calculated EA_a_ of the designed PDIs and PDI-Ph are tabulated in Table 1. It can be seen from Table 1 that the EA_a_ values for PDI-Si, PDI-N, PDI-P, PDI-O, PDI-S, and PDI-Se are larger than those of PDI-Ph, indicating better stability of designed PDIs with Lewis base heteroatoms. The result is also consistent with the larger EA_a_ corresponding to increased stability [53,54]. Furthermore, to express the resistance of chemical potential and the change in the number of electrons, absolute hardness (η) is calculated from the value of EA and the adiabatic ionization potential (IP_a_) (see Table 1). There is a small difference in the value of η in As-PDIs compared to that in PDI-Ph (<0.10 eV), suggesting their similar stability in the resistance of chemical potential. This investigation suggests that As-PDIs with a modified Lewis base heteroatom have adequate stability in the environment.

As stated in earlier studies, the good solubility of ETMs in a designative solution may improve the performance of PSCs, which can be fabricated in solution [26]. The solubility of the PDIs was evaluated through the solvation free energy (ΔG_solv_); lower ΔG_solv_ leads to larger solubility. The ΔG_solv_ was obtained from the energy variation of the PDIs in a particular solution and gas phase. It was reported experimentally that PDIs were usually investigated in solvents such as toluene and CH_2_Cl_2_, among others [35]. As a result, toluene was used here for the calculation of ΔG_solv_ of the PDIs. Better solubility of the material arises from lower ΔG_solv_. As shown in Table 1, the ∆G_solv_ for the new PDI material is 0.53, ~1.67 eV smaller than that of PDI-Ph, indicating better solubility with toluene.

### 3.3. Reorganization Energy, Transfer Integral, Transfer Rate, and Mobility

As stated in Marcus’ theory, electron reorganization energy (*λ*_electron_) is one of the most important parameters that is used to evaluate the electron transport rate (*k*_electron_) and mobility (*μ*_electron_) of ETMs. Lower reorganization energy corresponds to better transport performance. The good electron transfer ability of ETMs is vital for the improvement of PCE (power conversion efficiency) in PSCs. Here, reorganization energies were investigated for potential ETMs (PDI-Si, PDI-N, PDI-P, PDI-O, PDI-S, and PDI-Se) and available ETM (PDI-Ph), which are tabulated in Table 2. The *λ*_electron_ values for PDI-P, PDI-O, PDI-S, and PDI-Se are similar to that of PDI-Ph (with a deviation smaller than 0.02 eV), suggesting that asymmetrical geometry (versus symmetrical structure) has little effect on the variation of electron reorganization in PDIs. However, the values of PDI-Si and PDI-N have a greater difference (0.09 and 0.17 eV) with that of PDI-Ph, which is mainly ascribed to the introduction of silicon and nitrogen in As-PDIs. The hole reorganization energies were also obtained for related PDIs; these are shown in Supplementary Materials (Table S5). The small hole reorganization energies in these PDI derivatives demonstrate that the materials may have good hole transfer ability.

Other than electron reorganization energy, a key parameter to determine the electron transfer rate and mobility for ETMs is electron transfer integral (*V*_electron_), which is mainly dependent on the non-local electronic coupling and the relative position of neighboring molecules in electron transfer pathways. On the basis of the predicted crystal geometries, *V*_electron_ was calculated for the PDIs and provided in Table 2. As discussed in eqn (1), a larger value of *V*_electron_ will produce more improvement in electron transfer rate. Our calculated *V*_electron_ values for all designed PDIs are comparable to PDI-Ph (the deviation smaller than 0.08 eV), suggesting good electron transfer ability in As-PDIs. Additionally, the small difference in the value of *V*_electron_ in designed PDIs arises from the variation of the Lewis base heteroatom.

As listed in Table 2, *k*_electrons_ in PDI-Si, PDI-N, PDI-P, PDI-S, and PDI-Se have the same order of magnitude (10^13^ s^−1^) as that of PDI-Ph, while PDI-O has an even greater *k*_electron_ (1.58 × 10^14^ s^−1^), suggesting largely improved electron transfer ability of PDI-O. Correspondingly, high values of *μ*_electron_ (0.57~5.94 cm^2^ V^−1^ s^−1^) were found in the new PDIs, in the order of PDI-O > PDI-S > PDI-Se > PDI-P > PDI-N > PDI-Si. Excitingly, increased electron mobility was observed in PDI-O (5.94 cm^2^ V^−1^ s^−1^), PDI-S (3.52 cm^2^ V^−1^ s^−1^), and PDI-Se (3.06 cm^2^ V^−1^ s^−1^) compared to PDI-Ph (2.18 cm^2^ V^−1^ s^−1^). The varying electron mobility of these materials was mainly ascribed to the variation of reorganization energy, transfer integral, and centroid-to-centroid distance between adjacent molecules in the main pathway of electron transfer, which was strongly influenced by frontier molecular orbitals and crystal structure. The values of hole mobility (*μ*_hole_) indicate that most of the designed molecules also have good hole transportation performance, as shown in Table S5. The results suggest that the introduction of heteroatom-modified functional groups—especially the introduction of oxygen, sulfur, and selenium—with methyl in PDIs, to form asymmetrical geometries, has a great impact on the refinement of electron transfer properties.

### 3.4. Molecular Stacking

**Figure 4:**
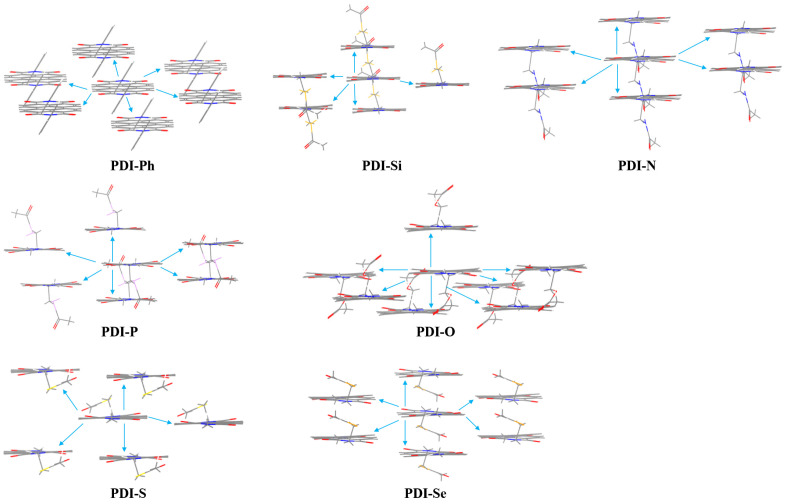
The predicted crystal structures and the possible electron-hopping pathways for the PDIs.

The crystal structures of proposed PDIs were investigated to explore the relative positions of molecules to understand electron transporting behavior. The packing modes of predicted crystal structures for the PDIs are shown in Figure 4. It is shown that 1D π-π stacking was found in the crystals of PDI-Ph, PDI-Si, PDI-N, and PDI-Se while 2D π-π stacking was found in the crystals of PDI-P, PDI-O, and PDI-S, for which the centroid-to-centroid distance between adjacent molecules in the main pathway of electron transfer was 3.86 Å (PDI-Ph), 4.56 Å (PDI-Si), 4.38 Å(PDI-N), 5.20 Å (PDI-P), 4.41 Å (PDI-O), 5.83 Å (PDI-S), and 4.33 Å (PDI-Se).

**Figure 5:**
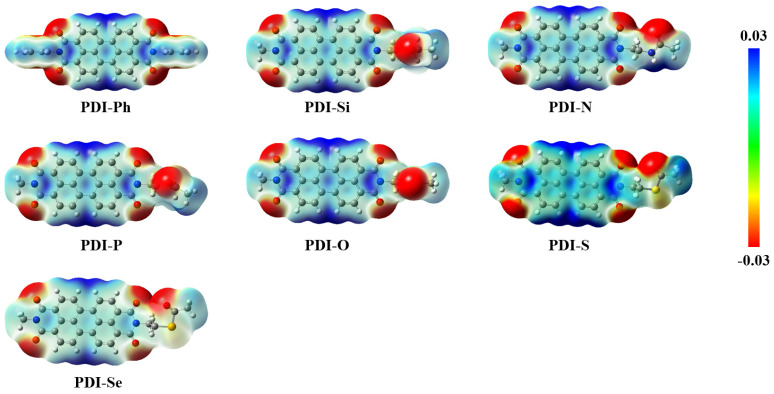
Electrostatic potential (ESP) mapped onto a surface of electron distribution for studied PDIs.

To analyze the variation in packing motifs caused by chemical modification, the electrostatic potential (ESP) maps for the PDIs were investigated as displayed in Figure 5. For PDI-Ph, the positive potentials were mainly localized around the hydrogen and nitrogen atoms in the PDI core while the negative potentials were primarily distributed on the oxygen atoms in PDI core. Additional negative potentials were found on the oxygen atoms in the functional branched groups of designed PDIs, for which stronger electrostatic attraction could be predicted. Maximum electrostatic attraction normally emerges between positive and negative potentials; then, π-π stacking of the crystal motifs in these PDIs was displayed, due to the hydrogen/nitrogen (in the PDI core)–oxygen (in PDI core) or the hydrogen/nitrogen (in PDI core)–oxygen (functional branched groups) attractions. In PDI-Ph, the interaction between PDI cores leads to 1D π-π stacking of the crystal geometry. Similarly, the negative potentials in functional branched chains of PDI-Si, PDI-N, and PDI-Se are adjacent to the PDI core, which makes the attraction between PDI cores stronger than the interaction of the PDI core with the side chain; then, 1D π-π stacking appears in the crystal motif. However, the larger distance from the negative part in the side chain to the PDI core exhibited in PDI-P and PDI-O, which drives the attraction between PDI cores, is comparable with the interaction of the PDI core and side chain. As a result, 2D π-π stacking is reasonable for the crystal structure of PDI-P and PDI-O. It is worth noting that 2D π-π stacking is also found in the crystal of PDI-S. This could be explained by the strong positive potentials at the end of the functional side chain, which attract the negative parts in the PDI core and side chain.

**Figure 6:**
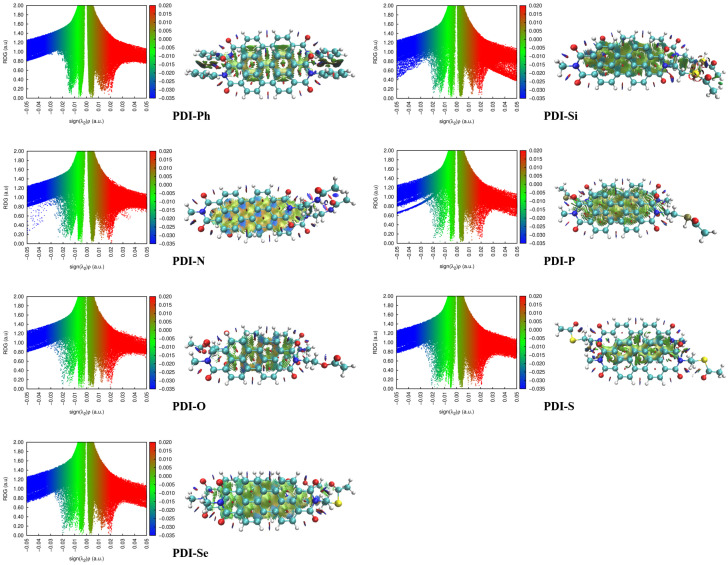
The 2D scatter plots and color-filled RDG isosurface for the dimers with the largest transfer integral for the PDIs.

For the purpose of obtaining more insight into the molecular packing style in the crystal, the non-covalent interaction in the dimers with the largest transfer integral of the studied PDIs was investigated by the analysis of reduced-density gradients (RDGs). As shown in Figure 6, the low-gradient spikes in the left column of each molecule appear near zero, while the isosurface images in the right column of each molecule are green. The large van der Waals interactions in PDI-Ph, PDI-Si, PDI-N, PDI-P, PDI-O, PDI-S, and PDI-Se lead to the crystal packing of π-π motifs. The non-covalent interaction is stronger in the following order: PDI-S < PDI-P < PDI-Si < PDI-O < PDI-N < PDI-Se < PDI-Ph, which is inverse to the intermolecular distance discussed earlier (molecular stacking).

### 3.5. Interaction Energy

**Figure 7:**
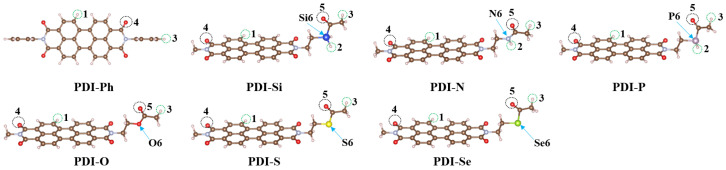
The sketch map of interactions between selected atoms in investigated molecules with I^−^, Pb^2+^, and MA^+^.

For the purpose of evaluating the interaction between these PDIs’ molecules and perovskite, the interaction energies of selected hydrogen, double-bonded oxygen, and different Lewis base heteroatoms (Si, N, P, O, S, or Se) in the PDIs with I^−^ (anionic iodine), Pb^2+^ (cationic lead), or MA^+^ (CH_3_NH_3_^+^) were calculated; values are tabulated in Table 3. The specific interaction pictures are shown in Figure 7 and Figures S2–S8. It could be observed from the obtained data that strong interactions appeared in the PDIs with I^−^, Pb^2+^, or MA^+^. It is worth noting that powerful attractions between hydrogen, double-bond oxygen, and heteroatoms in the side chain of As-PDIs with I^−^, Pb^2+^, or MA^+^ are detected. The additional hydrogen, double-bond oxygen, and heteroatoms in As-PDIs may increase their gravitation for I^−^, Pb^2+^, or MA^+^. In particular, the binding energy between heteroatom in As-PDIs and Pb^2+^ is in the following order: N-Pb^2+^ (−8.37 eV) > O-Pb^2+^ (−7.14 eV) > S-Pb^2+^ (−7.05 eV) > P-Pb^2+^ (−6.70 eV) > Se-Pb^2+^ (−6.68 eV) > Si-Pb^2+^ (−6.37 eV). This investigation may predict the good interaction of the studied PDIs, especially the designed ones, with perovskite, when they work as ETMs in PSCs.

### 3.6. Interface Property

The perovskite/ETM interface properties have significant influence on the parameters that evaluate the performance of PSCs, such as *V*oc (open circuit voltage), *J*sc (short circuit current density), *FF* (filling factor), and ultimate PCE. For the purpose of investigating perovskite/ETM properties, the (110) surface of tetragonal MAPbI_3_, which is normally used as one of the major facets in theoretical and experimental research, was employed here. As reported, the PbI_2_^−^- and MA^−^-terminations showed similar trend in the interface structure and charge transfer behavior; PbI_2_^−^-terminated surface was considered due to easier adsorption [55,56].

**Figure 8:**
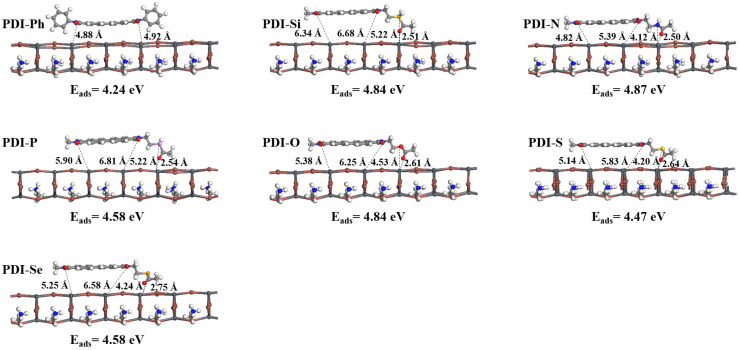
The optimized geometries of ETMs adsorbed on perovskite surface, together with the corresponding adsorption energy (Eads) and the key N…Pb, O…Pb, and S…Pb distances (dashed lines).

The geometries of the ETMs adsorbed on the perovskite surface were optimized and are shown in Figure 8. As shown in Figure 8, the PDI skeleton in As-PDIs is nearly parallel to the perovskite surface. For PDI-Ph, the N (in the PDI backbone) interacting with Pb (in the perovskite surface) leads to efficient adsorption of ETM on perovskite, with N…Pb distances of 4.88 and 4.92 Å, respectively. For designed PDIs, in addition to the interaction of N in the PDI backbone with Pb, additional interaction between the Lewis base heteroatom or double bond oxygen in the side chain and Pb in the perovskite surface were found. The heteroatom…Pb and double bond oxygen…Pb distances between new ETMs and perovskite surface, displayed in Figure 8, were 4.12~5.22 Å and 2.50~2.75 Å, respectively. Correspondingly, the structural modification in ETMs leads to the variation of adsorption energies (E_ads_), as listed in Figure 8. The larger Eads for designed ETMs than that of PDI-Ph indicated improved adsorption performance with the appearance of Lewis base heteroatoms Si, N, P, O, S, or Se in the molecular functional groups. The different E_ads_ of designed ETMs on perovskite surface (the largest variation of 0.40 eV) explained the influence of various heteroatoms on the interface interaction.

**Figure 9:**
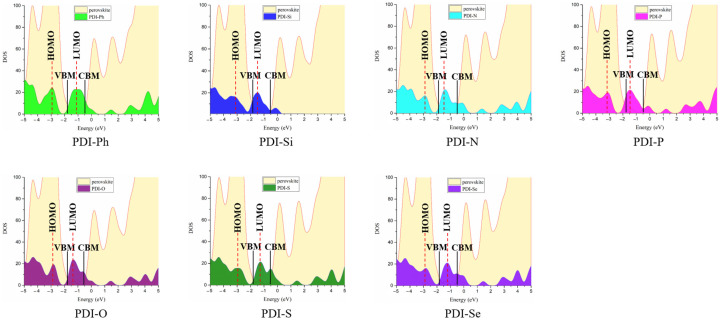
Density of states (DOS) of perovskite/ETMs with separate contributions from perovskite and ETMs (PDI-Ph, PDI-Si, PDI-N, PDI-P, PDI-O, PDI-S, and PDI-Se).

The perovskite/ETM interface properties were further investigated through the analysis of density of states (DOS), as shown in Figure 9. The DOS was composed of the contributions from perovskite and each ETM. It was found that the HOMO and LUMO energies of ETMs are lower than the VBM and CBM of perovskite, respectively. This may prevent the hole transfer and accelerate the electron extraction from the perovskite layer to the ETM layer. The recombination of hole and electron could be decreased, and electron transfer ability will be enhanced at the perovskite/ETM interface. Furthermore, it was observed that the electron overlaps between the CBM of perovskite and LUMO of designed As-PDIs are larger than those of perovskite/PDI-Ph, indicating enhanced motivation and better electron injection property from perovskite to new ETMs. This suggests that the As-PDIs with the introduction of a Lewis base heteroatom may strengthen interface electron extraction and improve device performance.

**Figure 10:**
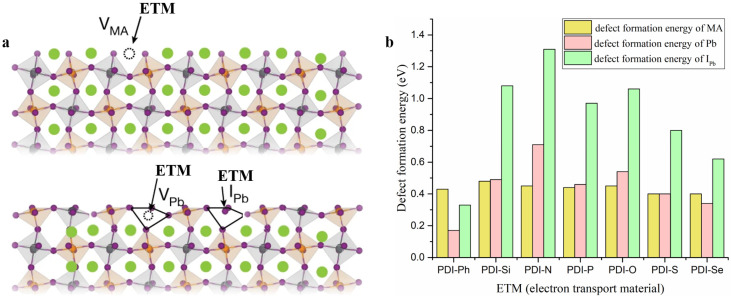
(**a**), Schematic diagram of the interaction between ETMs with acceptor-like defects—that is, methylammonium (MA) vacancy (V_MA_), Pb vacancy (V_Pb_), and I_Pb_ vacancy, (**b**)—values of defect formation energy of MA, Pb, and I_Pb_ in perovskite and ETM/perovskite.

It is known that the appearance of MA (CH_3_NH_3_^+^), Pb, or I_Pb_ defect (Figure 10a) may affect the ultimate performance of PSCs. Here, the defect formation energies of MA, Pb, and I_Pb_ were calculated, with PDIs adsorbed on perovskite. It is shown in Figure 10b that large defect formation energies were obtained in all perovskite/ETM interfaces. Moreover, with designed As-PDIs adsorbed on a perovskite surface, almost all the MA, Pb, and I_Pb_ defect formation energies were increased compared with perovskite/PDI-Ph, suggesting the better defect-passivation ability of new ETMs. Particularly, the defect deactivation ability of PDI-N was significantly increased.

In summary, the extensive analysis in the present work proved that designed As-PDIs with a Lewis base heteroatom have enhanced electron extraction capability, good electron transportation property (large electron mobility), and better defect deactivation ability at the perovskite/ETM interface, indicating improved interface performance for PSCs. Such results are encouraging and could be used as guidelines for the construction of efficient ETMs for high-performance PSCs.

## 4. Conclusions

Six novel PDI derivatives were successfully designed as ETMs for PSCs using state-of-the-art techniques. The proposed PDI molecules with asymmetrical geometries and introduction of Lewis base heteroatoms (Si, N, P, O, S, and Se) led to greatly improved performance as ETMs. The data obtained for these materials were compared with those of an available ETM (PDI-Ph). Quantum chemical investigations revealed that designed compounds have better stability and solubility, as well as more consistent HOMO and LUMO energy levels with perovskite. Strong molecular interactions among the heteroatoms and hydrogen atoms led to π-π stacking of crystal motifs in these ETMs. Greatly increased electron transfer performance was found in newly designed ETMs (PDI-O, PDI-S, and PDI-Se) compared to that of PDI-Ph, ascribed to the greater electron mobility. Among the designed asymmetrical molecules, PDI-O has the highest electron mobility (5.94 cm^2^ V^−1^ s^−1^). It is worth noting that Lewis base heteroatoms in As-PDIs greatly improve perovskite/ETMs’ interface interaction property. Moreover, enhanced passivation ability of MA, Pb, and I_Pb_ defects in perovskite was obtained in newly designed ETMs, especially in PDI-N. Thus, improved perovskite/ETM interface property and device performance could be realized by novel ETMs that have great potential for application in PSCs. The research in this contribution is of vital importance for the design of high-efficiency components for PSCs with the aim of promoting device performance.

## Figures and Tables

**Table 1:** The adiabatic ionization potential (IP_a_, eV), adiabatic electron affinity (EA_a_, eV), absolute hardness (η, eV), and solvation free energy (∆G_solv_, kcal mol^−1^) of PDIs.

PDIs	IP_a_	EA_a_	η	∆G_solv_
PDI-Ph	7.39	2.76	2.32	−6.15
PDI-Si	7.39	2.79	2.30	−7.82
PDI-N	7.47	3.03	2.22	−7.70
PDI-P	7.42	2.79	2.32	−6.68
PDI-O	7.52	2.82	2.35	−7.11
PDI-S	7.47	2.79	2.34	−6.81
PDI-Se	7.42	2.78	2.32	−7.19

**Table 2:** The electron reorganization energy *λ*_electron_ (eV), centroid-to-centroid distance (*d*, Å), electron transfer integral *V*_electron_ (eV), electron transfer rate *k*_electron_ (s^−1^), and electron mobility *μ*_electron_ (cm^2^ V^−1^ s^−1^) for studied molecules.

PDIs	*d*	*λ*_electron_	*V*_electron_	*k*_electron_	*μ*_electron_
PDI-Ph	3.86	0.27	0.18	7.56 × 10^13^	2.18
PDI-Si	4.56	0.36	0.13	1.42 × 10^13^	0.57
PDI-N	4.38	0.44	0.26	2.36 × 10^13^	0.88
PDI-P	5.20	0.29	0.15	4.17 × 10^13^	2.18
PDI-O	4.41	0.27	0.26	1.58 × 10^14^	5.94
PDI-S	5.83	0.29	0.17	5.36 × 10^13^	3.52
PDI-Se	4.33	0.27	0.19	8.43 × 10^13^	3.06

**Table 3:** Interaction energies (eV) between selected atoms (the atomic number could be found in Figure 7) in investigated molecules with I^−^, Pb^2+^, and MA^+^.

	H1-I^−^	H2-I^−^	H3-I^−^	O4-Pb^2+^	O5-Pb^2+^	O4-MA^+^	O5-MA^+^	X6-Pb^2+ a^
PDI-Ph	−1.91		−1.86	−7.83		−1.26		
PDI-Si	−1.53	−1.56	−1.55	−6.60	−6.33	−1.32	−1.16	−6.37
PDI-N	−1.90	−2.04	−2.04	−7.09	−8.40	−1.27	−1.69	−8.37
PDI-P	−1.66	−1.69	−1.65	−6.84	−7.76	−1.22	−1.17	−6.70
PDI-O	−1.89		−1.85	−6.79	−6.86	−1.15	−1.20	−7.14
PDI-S	−1.35		−1.34	−6.49	−7.40	−1.22	−1.80	−7.05
PDI-Se	−1.64		−1.66	−6.99	−7.70	−1.19	−1.64	−6.68

^a^ X = Si, N, P, O, S, and Se.

## Data Availability

Not applicable.

## Supplementary Materials

The following supporting information can be downloaded at: https://www.mdpi.com/article/10.3390/nano13091560/s1, Figure S1: TD-DFT UV visible absorption spectrum for investigated molecules; Figure S2: Interactions between H or O in PDI-Ph and I^−^, Pb^2+^, or MA^+^; Figure S3: Interactions between H, O, or Si in PDI-Si and I^−^, Pb^2+^, or MA^+^; Figure S4: Interactions between H, O, or N in PDI-N and I^−^, Pb^2+^, or MA^+^; Figure S5: Interactions between H, O, or P in PDI-P and I^−^, Pb^2+^ or MA^+^; Figure S6: Interactions between H or O in PDI-O with I^−^, Pb^2+^, or MA^+^; Figure S7: Interactions between H, O, or S in PDI-S and I^−^, Pb^2+^, or MA^+^; Figure S8: Interactions between H, O, or Se in PDI-Se and I^−^, Pb^2+^, or MA^+^; Table S1: The name and abbreviation of investigated molecules; Table S2: Optimized geometrical parameters and frequencies for studied molecules; Table S3: The highest occupied molecular orbital (HOMO) and lowest unoccupied molecular orbital (LUMO) energies of PDI-Ph calculated by different methods together with 6-31+G(d,p) basis set; Table S4: Absorption maximum wavelength (λ_max_), oscillator strength, excitation energy, electronic transition, and main configuration for the PDI derivatives; Table S5: The hole reorganization energy *λ*_hole_ (eV), centroid-to-centroid distance (*d*, Å), hole transfer integral *V*_hole_ (eV), hole transfer rate *k*_hole_ (s^−1^), and hole mobility *μ*_hole_ (cm^2^ V^−1^ s^−1^) for studied molecules. File S1: Vibrational frequencies of PDI-Ph; File S2: Geometrical parameters of PDI-Si; File S3: Vibrational frequencies of PDI-Si; File S4: Geometrical parameters of PDI-N; File S5: Vibrational frequencies of PDI-N; File S6: Geometrical parameters of PDI-P; File S7: Vibrational frequencies of PDI-P; File S8: Geometrical parameters of PDI-O; File S9: Vibrational frequencies of PDI-O; File S10: Geometrical parameters of PDI-S; File S11: Vibrational frequencies of PDI-S; File S12: Geometrical parameters of PDI-Se; File S13: Vibrational frequencies of PDI-Se.
