# Supplementary material for: Rational Design of Lewis Base Electron Transport Materials for Improved Interface Property in Inverted Perovskite Solar Cells: A Theoretical Investigation
Source: Nanomaterials (Basel). 2023 May 5;13(9):1560. doi: 10.3390/nano13091560 (PMC10180708; doi:10.3390/nano13091560)
Supplement: Supplementary file 1 [file nanomaterials-13-01560-s001.zip › nanomaterials-2365079-supplementary.pdf]

nanomaterials

Article

Rational Design of Lewis Base Electron Transport Materials for Improved Interface Property in Inverted Perovskite Solar Cells: A Theoretical Investigation

Xueqin Ran [1], Jixuan Yang [1], Mohamad Akbar Ali [2,3,*], Lei Yang [4,*] and Yonghua Chen [1]

[1] Key Laboratory of Flexible Electronics (KLOFE) & Institution of Advanced Materials (IAM), School of Flexible Electronics (Future Technologies), Nanjing Tech University (Nanjing Tech), Nanjing 211816, China; 202161122151@njtech.edu.cn (J.Y.)
[2] Centre for Molecular Systems and Organic Devices (CMSOD), Key Laboratory for Organic Electronics and Information Displays & Jiangsu Key Laboratory for Biosensors, Institute of Advanced Materials (IAM), Jiangsu National Synergetic Innovation Center for Advanced Materials (SICAM), Nanjing University of Posts & Telecommunications, 9 Wenyuan Road, Nanjing 210023, China
[3] Advanced Materials Chemistry Center (AMCC), Khalifa University of Science and Technology, Abu Dhabi P.O. Box 127788, United Arab Emirates
[4] Department of Chemistry, College of Art and Science, Khalifa University of Science and Technology, Abu Dhabi P.O. Box 127788, United Arab Emirates
* Correspondence: akbar.mohamad@ku.ac.ae (M.A.A.); iamlyang@njupt.edu.cn (L.Y.); Tel.: +971-23124159 (M.A.A.); +86-025-8586-6999 (L.Y.)

Table S1. The name and abbreviation of investigated molecules.

Molecular Name	Abbreviation
2,9-diphenylanthra[2,1,9-*def*:6,5,10-*d′e′f′*]diisoquinoline-1,3,8,10(2*H*,9*H*)-tetraone	PDI-Ph
2-(2-(acetylsilyl)ethyl)-9-methylanthra[2,1,9-*def*:6,5,10-*d′e′f′*]diisoquino-line-1,3,8,10(2*H*,9*H*)-tetraone	PDI-Si
N-(2-(9-methyl-1,3,8,10-tetraoxo-3,8,9,10-tetrahydroanthra[2,1,9-*def*:6,5,10-*d′e′f′*]diisoquinolin-2(1*H*)-yl)ethyl)acetamide	PDI-N
2-(2-(acetylphosphaneyl)ethyl)-9-methylanthra[2,1,9-*def*:6,5,10-*d′e′f′*]diisoquinoline-1,3,8,10(2*H*,9*H*)-tetraone	PDI-P
2-(9-methyl-1,3,8,10-tetraoxo-3,8,9,10-tetrahydroanthra[2,1,9-*def*:6,5,10-*d′e′f′*]diisoquinolin-2(1*H*)-yl)ethyl acetate	PDI-O
S-(2-(9-methyl-1,3,8,10-tetraoxo-3,8,9,10-tetrahydroanthra[2,1,9-*def*:6,5,10-*d′e′f′*]diisoquinolin-2(1*H*)-yl)ethyl) ethanethioate	PDI-S
Se-(2-(9-methyl-1,3,8,10-tetraoxo-3,8,9,10-tetrahydroanthra[2,1,9-*def*:6,5,10-*d′e′f′*]diisoquinolin-2(1*H*)-yl)ethyl) ethaneselenoate	PDI-Se

Table S2. Optimized geometrical parameters and frequencies for studied molecules. 3-1. Geometrical parameters of PDI-Ph.

Center	Atomic	Atomic	Coordinates (Angstroms)		
Number	Number	Type	X	Y	Z
1	6	0	−5.060451	−1.249590	0.000315

2	6	0	–5.060451	1.249589	0.000475
3	7	0	–5.712114	0.000000	0.000335
4	6	0	–1.481160	-2.431583	0.000013
5	6	0	–0.735848	–1.249806	–0.000026
6	6	0	–1.434033	0.000000	0.000062
7	6	0	–2.865335	0.000000	0.000196
8	6	0	–3.576710	–1.225853	0.000223
9	6	0	–2.880946	–2.423793	0.000123
10	6	0	–0.735848	1.249806	0.000017
11	6	0	–3.576710	1.225853	0.000283
12	1	0	–3.441087	–3.352657	0.000140
13	6	0	–2.880946	2.423793	0.000210
14	6	0	–1.481160	2.431583	0.000089
15	1	0	–3.441087	3.352657	0.000278
16	8	0	–5.701258	2.290843	0.000162
17	8	0	–5.701258	–2.290843	0.000115
18	6	0	2.880946	–2.423793	–0.000354
19	6	0	3.576710	–1.225853	–0.000422
20	6	0	2.865335	0.000000	–0.000337
21	6	0	1.434033	0.000000	–0.000205
22	6	0	0.735848	–1.249806	–0.000163
23	6	0	1.481160	–2.431583	–0.000242
24	1	0	3.441087	–3.352657	–0.000416
25	6	0	3.576710	1.225853	–0.000350
26	6	0	0.735848	1.249806	–0.000106
27	6	0	1.481160	2.431583	–0.000113
28	6	0	2.880946	2.423793	–0.000217
29	1	0	3.441087	3.352657	–0.000220

30	6	0	5.060451	1.249590	–0.000483
31	6	0	5.060451	–1.249589	–0.000570
32	7	0	5.712114	0.000000	–0.000400
33	8	0	5.701258	2.290843	–0.000038
34	8	0	5.701258	–2.290843	–0.000161
35	6	0	–9.245519	0.000159	–1.210074
36	6	0	–7.849487	0.000158	–1.212337
37	6	0	–7.161781	0.000000	0.000196
38	6	0	–7.849715	–0.000158	1.212600
39	6	0	–9.245746	–0.000159	1.210072
40	6	0	–9.944657	0.000000	–0.000066
41	1	0	–9.784954	0.000284	–2.152424
42	1	0	–7.295420	0.000281	–2.145637
43	1	0	–7.295824	–0.000281	2.146004
44	1	0	–9.785358	–0.000284	2.152321
45	6	0	7.849842	0.000006	–1.212441
46	6	0	9.245872	0.000005	–1.209767
47	6	0	9.944657	0.000000	0.000444
48	6	0	9.245393	-0.000005	1.210379
49	6	0	7.849361	-0.000006	1.212496
50	6	0	7.161781	0.000000	–0.000109
51	1	0	7.296048	0.000009	–2.145903
52	1	0	9.785583	0.000009	–2.151960
53	1	0	9.784729	–0.000009	2.152785
54	1	0	7.295196	–0.000009	2.145737
55	1	0	0.982058	–3.392593	–0.000212
56	1	0	–0.982058	3.392592	0.000054
57	1	0	0.982058	3.392592	–0.000026

58	1	0	−0.982058	−3.392593	−0.000055
59	1	0	11.030642	0.000000	0.000660
60	1	0	−11.030642	0.000000	−0.000168

3-1. Vibrational frequencies of PDI-Ph.

15.502700 22.809800 27.788200 30.722600 31.552700 46.565900 57.594400 65.750400 71.903700 84.161900 98.666300 126.401600 127.122100 138.117600 152.243600 179.506400 185.791300 218.089600 233.011500 233.659200 271.885000 272.696900 289.889200 302.890000 314.187700 346.577400 351.010000 387.416400 390.913100 420.553200 420.565000 430.656000 436.614000 437.413000 439.941300 446.802700 458.200400 478.002600 480.357000 481.142600 505.921400 518.108600 519.716600 530.458800 533.156100 546.992500 576.061700 601.830300 620.094700 623.915000 629.964100 632.426300 643.242100 648.701600 649.059700 655.249600 694.059500 704.198100 707.871700 712.241700 714.550700 721.799000 735.185900 754.713500 755.310500 762.902100 768.893200 771.327300 776.051200 802.884800 821.740100 827.056400 843.072400 845.836700 845.837100 853.006100 861.901600 863.827300 865.062500 865.367900 904.778100 927.308300 940.181500 970.562400 976.381600 976.383900 987.446800 990.137800 991.403600 994.706100 998.384400 1002.147800 1002.362200 1018.789300 1018.836800 1047.636600 1048.993800 1066.162400 1079.868200 1101.506300 1101.509700 1150.296700 1157.848100 1165.413800 1167.343900 1168.057900 1184.241500 1184.242300 1197.430400 1197.452600 1203.754500 1207.906000 1224.456100 1228.544600 1249.918500 1257.169700 1268.977700 1276.107500 1317.797700 1323.653300 1334.018200 1336.509600 1336.514100 1346.367900 1355.946600 1355.947600 1366.968700 1380.970600 1382.331500 1395.434400 1416.362200 1433.103200 1457.084400 1477.516700 1487.768100 1487.769500 1494.513900 1510.071100 1531.718200 1531.771400 1549.800700 1565.432300 1609.220600 1619.526900 1626.033200 1635.957500 1636.469900 1641.868700 1641.869600 1649.408800 1649.422900 1653.699600 1724.787500 1724.811300 1758.076600 1762.648300 3184.126500 3184.129800 3193.100600 3193.101100 3201.444600 3201.463900 3208.569000 3208.572900 3213.447300 3213.512200 3213.521100 3213.589500 3218.662900 3218.788100 3226.887900 3226.949300 3238.492300 3238.566000

3-2. Geometrical parameters of PDI-Si.

Center Number	Atomic Number	Atomic Type	Coordinates (Angstroms) X	Y	Z
1	6	0	−3.237346	1.236973	−0.872257
2	6	0	−3.235718	−1.246059	−0.867757
3	7	0	−3.877985	−0.005140	−0.981855
4	6	0	0.306844	2.430365	−0.393093
5	6	0	1.047646	1.249492	−0.297530
6	6	0	0.357108	−0.001267	−0.387652
7	6	0	−1.061170	−0.002570	−0.576371
8	6	0	−1.768225	1.221756	−0.671306
9	6	0	−1.080916	2.420900	−0.577132
10	6	0	1.049637	−1.250727	−0.294396
11	6	0	−1.766574	−1.228157	−0.667198
12	1	0	−1.637165	3.349281	−0.649275
13	6	0	−1.077507	−2.425988	−0.569147
14	6	0	0.310356	−2.432904	−0.385723
15	1	0	−1.632462	−3.355405	−0.637880
16	8	0	−3.875445	−2.290432	−0.938710
17	8	0	−3.878415	2.280256	−0.947190
18	6	0	4.634824	2.425756	0.163459
19	6	0	5.325599	1.228122	0.252210
20	6	0	4.619339	0.002892	0.162444
21	6	0	3.200069	0.001460	−0.018665
22	6	0	2.507059	1.250769	−0.108330
23	6	0	3.246227	2.432756	−0.014754
24	1	0	5.189459	3.355168	0.234017
25	6	0	5.326537	−1.221427	0.252438

26	6	0	2.509284	−1.249387	−0.106610
27	6	0	3.250370	−2.430230	−0.012788
28	6	0	4.639134	−2.420765	0.164595
29	1	0	5.195926	−3.348981	0.235238
30	6	0	6.796892	−1.233345	0.439886
31	6	0	6.797546	1.250336	0.440414
32	7	0	7.442374	0.006547	0.521288
33	8	0	7.444351	−2.271611	0.523023
34	8	0	7.427725	2.298879	0.521969
35	1	0	2.751043	3.393608	−0.080056
36	1	0	0.805072	−3.393616	−0.315142
37	1	0	2.756568	−3.391843	−0.077419
38	1	0	0.800246	3.391975	−0.325585
39	6	0	8.899737	−0.024691	0.707834
40	6	0	−5.341644	−0.006309	−1.199250
41	1	0	9.364546	−0.564212	−0.119982
42	1	0	−5.563691	0.883052	−1.790060
43	1	0	9.252824	1.002855	0.742963
44	1	0	9.140703	−0.546203	1.636660
45	1	0	−5.563041	−0.899246	−1.784868
46	6	0	−6.130739	−0.002711	0.116549
47	1	0	−5.868258	−0.882711	0.715005
48	1	0	−5.867733	0.880252	0.710405
49	1	0	−8.475795	−1.206448	−0.854113
50	6	0	−8.854064	0.004911	1.641224
51	8	0	−8.149318	0.003209	2.641574
52	6	0	−10.367104	0.012508	1.743391
53	1	0	−10.771817	0.895953	1.233659

54	1	0	−10.781214	−0.865596	1.232020
55	1	0	−10.678102	0.013326	2.791533
56	14	0	−8.004593	−0.002448	−0.114375
57	1	0	−8.473919	1.196068	−0.864149

3-3. Vibrational frequencies of PDI-Si.

12.174200 15.401700 16.833000 27.856900 37.374800 42.484000 64.670800 70.136500 73.486400 78.780000 88.270800 95.231100 111.990000 122.691400 126.637700 127.177900 161.455900 168.265200 185.575200 199.890300 232.602200 236.002100 261.737800 273.338800 276.735400 316.051200 323.396200 343.493700 349.591700 363.582600 377.280800 383.917300 393.041300 396.530500 411.897500 427.431300 434.769800 442.560300 446.753500 457.296700 468.951800 478.334300 482.146700 515.169000 518.229000 530.900400 543.656500 575.918800 580.967700 596.087600 599.797900 616.482900 618.275400 624.873100 648.766600 655.242100 658.058800 687.805100 704.370100 716.057200 724.060200 729.393100 749.315700 750.685200 756.171300 759.136100 766.400200 769.026500 801.838700 803.045000 808.344100 832.095300 853.688900 863.487800 864.816100 865.351700 882.412800 920.016200 943.262300 945.140700 955.338000 973.710500 988.446500 990.225000 990.968200 995.638700 998.631400 1000.686200 1023.890700 1038.330900 1054.243000 1079.907100 1103.731600 1129.826300 1152.774700 1155.033700 1156.193600 1177.210700 1189.170800 1210.820300 1215.195400 1225.055000 1236.053200 1243.320500 1254.110700 1265.450900 1287.446000 1310.575400 1314.735100 1322.598700 1331.683300 1348.144200 1364.490400 1380.175000 1380.400400 1381.471200 1392.280900 1398.021300 1412.036700 1422.928300 1432.660800 1446.989800 1461.881400 1462.808300 1464.882900 1470.948200 1476.227900 1482.144700 1494.456800 1499.958300 1507.459200 1510.041800 1549.620200 1565.263800 1608.707800 1619.288800 1626.034600 1635.683000 1636.273000 1653.081500 1709.571100 1713.412600 1733.266200 1746.888800 1752.400500 2212.077800 2216.428200 3029.370800 3056.050500 3076.794100 3093.870500 3105.257000 3106.044600 3138.605000 3142.965600 3161.127900 3202.549600 3212.411100 3212.612200 3218.783500 3219.228300 3226.219600 3226.533500 3237.458900 3237.560900

3-4. Geometrical parameters of PDI-N.

Center Number	Atomic Number	Atomic Type	Coordinates (Angstroms) X	Y	Z
1	6	0	−3.542377	1.348345	−0.482429
2	6	0	−3.580355	−1.136630	−0.551628
3	7	0	−4.211693	0.120312	−0.571616
4	6	0	0.041723	2.478618	−0.192059
5	6	0	0.767409	1.284763	−0.164578
6	6	0	0.052978	0.046852	−0.245992
7	6	0	−1.373540	0.070728	−0.353639
8	6	0	−2.065048	1.307492	−0.376230
9	6	0	−1.354103	2.493956	−0.295400
10	6	0	0.729862	−1.214486	−0.222923
11	6	0	−2.102053	-1.141652	−0.436390
12	1	0	−1.898348	3.432016	−0.313199
13	6	0	−1.427192	−2.351488	−0.411245
14	6	0	−0.031707	−2.383079	−0.305737
15	1	0	−1.999724	−3.270621	−0.474262
16	8	0	−4.235779	−2.167639	−0.629574
17	8	0	−4.171940	2.402680	−0.493837
18	6	0	4.391707	2.396926	0.136701
19	6	0	5.066818	1.187402	0.162120
20	6	0	4.337545	−0.024711	0.078893
21	6	0	2.911237	−0.000961	−0.029962
22	6	0	2.234549	1.260187	−0.053635
23	6	0	2.996146	2.428614	0.029852
24	1	0	4.963886	3.316076	0.201295
25	6	0	5.028871	−1.261101	0.103798

26	6	0	2.197059	−1.238947	−0.112139
27	6	0	2.923061	−2.432446	−0.084533
28	6	0	4.319043	−2.447687	0.022026
29	1	0	4.863840	−3.385435	0.042157
30	6	0	6.506436	−1.299059	0.217492
31	6	0	6.546499	1.183463	0.278090
32	7	0	7.174694	−0.071153	0.298059
33	8	0	7.140509	−2.348410	0.242524
34	8	0	7.196017	2.220541	0.353518
35	1	0	2.513660	3.398015	0.013705
36	1	0	0.450854	−3.352394	−0.289258
37	1	0	2.411366	−3.384827	−0.146087
38	1	0	0.553789	3.430834	−0.131503
39	6	0	8.638752	−0.128558	0.412548
40	6	0	−5.679656	0.160593	−0.695402
41	1	0	9.054929	−0.649473	−0.452264
42	1	0	−5.925844	1.015613	−1.327319
43	1	0	9.009164	0.892344	0.460466
44	1	0	8.915674	−0.681160	1.312885
45	1	0	−5.995333	−0.764125	−1.176881
46	6	0	−6.362999	0.305568	0.677795
47	1	0	−6.265512	−0.621255	1.246609
48	1	0	−5.894159	1.122083	1.235811
49	7	0	−7.783885	0.578907	0.535849
50	1	0	−8.063260	1.506206	0.250490
51	6	0	−8.759386	−0.350117	0.792522
52	8	0	−8.505213	−1.492664	1.160619
53	6	0	−10.189153	0.127737	0.597682

54	1	0	−10.725916	0.017568	1.544130
55	1	0	−10.266958	1.165900	0.261613
56	1	0	−10.677428	−0.521828	−0.134045

3-5. Vibrational frequencies of PDI-N.

10.886400 19.162200 22.789300 27.611900 41.951900 46.351100 60.761000 66.486900 73.394800 82.736800 85.419500 100.843000 120.347200 124.140900 128.630000 162.823400 180.027000 194.914400 215.174700 235.590300 266.555400 272.804600 279.041900 287.709800 314.973300 324.822200 349.048000 364.385000 380.710700 389.174000 393.236300 396.852700 411.930400 423.484700 434.956100 442.816300 446.552200 463.918500 467.414000 475.908200 478.268000 501.137700 518.638500 530.426300 546.692000 576.003300 586.575500 598.300200 614.771800 618.393400 624.722100 629.516100 645.748100 652.458100 657.329500 688.046300 706.111700 717.416400 725.566300 731.372300 754.304900 757.359500 760.633900 764.554300 769.112000 805.518100 807.511400 832.093800 852.824000 862.783200 864.368000 865.308000 877.896600 906.889800 923.958000 966.299200 982.187700 988.155100 990.688000 995.520400 998.643800 998.738100 1032.974000 1042.598300 1050.624100 1055.722800 1078.438900 1087.227000 1105.484500 1114.078200 1152.164900 1156.563900 1171.779700 1182.373000 1204.700500 1212.354500 1218.787200 1227.611400 1244.930700 1254.981100 1266.083000 1272.566800 1311.049100 1315.346800 1319.454800 1323.513000 1332.148100 1347.791000 1353.408500 1369.146800 1376.221500 1383.451300 1385.144200 1395.818500 1403.928600 1414.353200 1424.991100 1432.868000 1446.891300 1464.699100 1473.857300 1476.068700 1481.402700 1489.282900 1492.029800 1494.756200 1499.817300 1507.166300 1510.372700 1548.565700 1549.911300 1565.547600 1609.128700 1619.314200 1626.319600 1635.809800 1636.646300 1653.148200 1711.531400 1713.797400 1748.462300 1752.289000 1757.930300 3054.418500 3070.560900 3076.940000 3104.622800 3133.124500 3136.721100 3141.598100 3143.198000 3174.546700 3202.653700 3213.106400 3213.304300 3219.027400 3219.242800 3226.755000 3226.883400 3238.074500 3238.266300 3641.598900

3-6. Geometrical parameters of PDI-P.

Center Atomic Atomic Coordinates (Angstroms)

Number	Number	Type	X	Y	Z
1	6	0	−3.281409	1.214022	−0.877563
2	6	0	−3.267969	−1.270808	−0.879346
3	7	0	−3.916991	−0.030783	−0.986909
4	6	0	0.257695	2.422777	−0.403266
5	6	0	1.003621	1.245042	−0.308074
6	6	0	0.318772	−0.008750	−0.399808
7	6	0	−1.099556	−0.016348	−0.588388
8	6	0	−1.812116	1.205071	−0.681270
9	6	0	−1.129998	2.407245	−0.586598
10	6	0	1.017073	−1.255146	−0.307754
11	6	0	-1.799004	−1.245212	−0.680605
12	1	0	-1.690263	3.333299	−0.657498
13	6	0	−1.104350	−2.440035	−0.584537
14	6	0	0.283443	−2.440677	−0.401124
15	1	0	−1.655046	−3.371906	−0.654333
16	8	0	−3.902829	−2.316873	−0.953270
17	8	0	−3.928921	2.254005	−0.947122
18	6	0	4.584564	2.437170	0.160649
19	6	0	5.280573	1.242597	0.249214
20	6	0	4.580183	0.014263	0.156226
21	6	0	3.161319	0.006540	−0.027682
22	6	0	2.462776	1.252742	−0.116966
23	6	0	3.196286	2.437991	−0.020368
24	1	0	5.134793	3.368999	0.233730
25	6	0	5.292862	−1.206880	0.246008
26	6	0	2.476493	−1.247337	−0.118442

27	6	0	3.222853	−2.424868	−0.025084
28	6	0	4.611262	−2.409253	0.154944
29	1	0	5.172152	−3.335012	0.225346
30	6	0	6.762891	−1.212245	0.436899
31	6	0	6.752094	1.271324	0.440911
32	7	0	7.402458	0.030511	0.521555
33	8	0	7.414825	−2.247610	0.520201
34	8	0	7.376992	2.322746	0.525485
35	1	0	2.696835	3.396663	−0.085163
36	1	0	0.782564	−3.399159	−0.331682
37	1	0	2.733677	−3.388687	−0.091955
38	1	0	0.746883	3.386496	−0.335363
39	6	0	8.859517	0.005718	0.711751
40	6	0	−5.378377	−0.036623	−1.201813
41	1	0	9.328782	−0.531078	−0.115303
42	1	0	−5.606835	0.841312	−1.807359
43	1	0	9.207890	1.034809	0.748552
44	1	0	9.100484	−0.515394	1.640803
45	1	0	−5.612709	−0.941564	−1.762832
46	6	0	−6.147661	−0.005683	0.124319
47	1	0	−5.908786	−0.891246	0.720983
48	1	0	−5.875346	0.880315	0.703832
49	1	0	−8.261544	1.308531	−0.391110
50	6	0	−8.573953	−0.075962	1.643137
51	8	0	−7.922940	−0.637356	2.505089
52	6	0	−9.914682	0.571664	1.935117
53	1	0	−9.781019	1.658785	1.994412
54	1	0	−10.636789	0.372023	1.136479

55	1	0	−10.298120	0.208184	2.891464
56	15	0	−7.997606	−0.069522	−0.154101

3-7. Vibrational frequencies of PDI-P.

14.008700 14.927200 27.508500 30.874300 38.056100 52.523800 66.878200 72.343300 76.394400
85.750100 89.285800 97.534400 102.157400 123.136600 127.196400 136.464700 162.669300 174.137900
190.515500 207.502500 236.338700 240.234700 269.062900 273.451400 299.783700 315.926800
324.136600 350.041000 364.113300 379.161200 385.403500 393.059800 396.633200 410.397700
411.916700 434.866800 442.581200 446.916400 457.443700 468.147600 478.324100 481.595600
507.707500 517.957500 531.082500 543.697300 575.880400 581.328600 597.962300 603.428400
618.295500 618.403800 624.939800 648.651000 658.062900 684.065700 689.986900 705.950100
716.289600 724.215400 730.244600 750.798800 755.183400 758.912000 763.229800 766.527100
775.384200 796.001900 806.825900 831.049400 834.313900 853.742000 863.559300 864.846700
865.465900 891.290300 921.023700 949.596500 964.310200 976.944800 988.605400 991.028400
995.750400 998.712400 1019.491800 1027.522000 1035.649600 1046.762300 1057.962600 1080.210200
1104.492800 1134.010400 1144.371800 1152.800500 1156.342800 1177.294200 1189.315400 1210.964900
1215.253000 1226.017300 1243.707700 1253.995200 1255.208400 1266.076000 1287.956800 1310.806600
1314.979200 1322.572800 1331.880800 1348.027600 1365.411700 1380.421800 1381.234900 1385.653400
1391.500400 1393.759400 1412.239600 1422.866100 1432.628300 1447.156300 1465.032000 1465.300000
1472.472100 1477.399200 1478.007800 1482.108000 1494.457500 1499.924800 1507.455000 1510.052200
1549.680500 1565.295500 1608.691100 1619.289900 1626.107900 1635.682000 1636.274500 1653.126300
1711.116300 1713.568300 1747.704000 1752.525800 1755.245500 2387.993700 3040.540200 3076.072700
3076.869300 3107.934500 3109.501800 3132.571300 3143.086900 3147.720000 3166.084800 3202.621700
3212.551800 3212.692000 3218.955800 3219.241700 3226.378500 3226.553800 3237.573800 3237.724300

3-8. Geometrical parameters of PDI-O.

Center	Atomic	Atomic	Coordinates (Angstroms)		
Number	Number	Type	X	Y	Z

1	6	0	–3.567420	1.241208	–0.653463
2	6	0	–3.566217	–1.246972	–0.649907
3	7	0	–4.213846	–0.003292	–0.734084
4	6	0	–0.008992	2.431390	–0.303586
5	6	0	0.734003	1.249959	–0.234283
6	6	0	0.040197	–0.000600	–0.300581
7	6	0	–1.384016	–0.001512	–0.438628
8	6	0	–2.093082	1.223530	–0.507270
9	6	0	–1.402162	2.422574	–0.437666
10	6	0	0.735540	–1.250242	–0.232434
11	6	0	–2.091860	–1.227425	–0.504180
12	1	0	-1.960052	3.351330	-0.489374
13	6	0	–1.399609	–2.425539	–0.431735
14	6	0	–0.006351	–2.432575	–0.298417
15	1	0	–1.956533	–3.355024	–0.480739
16	8	0	–4.209751	–2.289144	–0.700278
17	8	0	–4.211936	2.282617	–0.707076
18	6	0	4.334878	2.425229	0.106110
19	6	0	5.027741	1.227374	0.171262
20	6	0	4.318613	0.002398	0.104136
21	6	0	2.894228	0.001352	–0.029614
22	6	0	2.198969	1.250775	–0.095111
23	6	0	2.941038	2.432557	–0.025764
24	1	0	4.891736	3.354484	0.158808
25	6	0	5.028211	–1.222045	0.169673
26	6	0	2.200719	–1.249231	–0.095011
27	6	0	2.944248	–2.430265	–0.027241

28	6	0	4.338248	−2.421228	0.103642
29	1	0	4.896886	−3.349593	0.154946
30	6	0	6.504200	−1.234316	0.309021
31	6	0	6.505329	1.249134	0.311482
32	7	0	7.152251	0.005287	0.370493
33	8	0	7.153255	-2.272924	0.370119
34	8	0	7.137491	2.297648	0.373313
35	1	0	2.444286	3.393620	−0.073659
36	1	0	0.490536	−3.393282	−0.246221
37	1	0	2.448503	−3.391818	−0.075854
38	1	0	0.486927	3.392718	−0.253642
39	6	0	8.615012	−0.026239	0.509845
40	6	0	−5.674182	−0.004141	−0.923317
41	1	0	9.052505	−0.566824	−0.331995
42	1	0	−5.934188	0.888456	-1.491982
43	1	0	8.969310	1.001222	0.532445
44	1	0	8.885676	−0.546736	1.431023
45	1	0	−5.933659	−0.899570	−1.487736
46	6	0	−6.400345	−0.001096	0.419381
47	1	0	−6.158027	−0.890277	1.007472
48	1	0	−6.158056	0.890749	1.003420
49	6	0	−8.658147	0.001893	1.164673
50	8	0	−8.281652	0.005200	2.318118
51	6	0	−10.095666	0.001921	0.708244
52	1	0	−10.293337	0.885839	0.094168
53	1	0	−10.292439	−0.878360	0.088798
54	1	0	−10.751389	−0.000673	1.578537
55	8	0	−7.809441	−0.001825	0.107199

3-9. Vibrational frequencies of PDI-O.

14.885300 15.978800 25.449600 28.800600 39.966700 46.333500 65.835600 70.418000 74.184800 82.580000 86.643300 106.987300 123.266400 127.575000 150.661800 163.393500 178.120900 183.735300 194.111200 224.751300 237.099900 265.249300 273.001500 278.589500 315.627700 324.690700 351.276300 364.459600 381.736400 386.663600 393.087300 396.479300 411.756200 417.596400 435.053600 442.742700 447.109600 464.335900 475.006000 478.078400 497.176500 518.073200 531.012700 546.747000 576.095800 587.403500 598.599800 602.901400 618.487700 624.972500 631.722100 643.587500 650.657400 657.633600 688.424900 706.207800 717.367700 725.899200 731.676900 754.451800 758.087600 764.567000 765.348000 774.193300 806.785100 812.401500 831.748700 853.390700 863.228000 864.785100 865.356700 890.599100 902.463800 929.372500 963.812200 986.261200 987.524400 988.527900 990.935100 995.763000 998.683200 1038.562100 1048.522900 1065.468700 1070.001900 1077.988600 1086.106500 1092.146900 1106.834700 1152.369000 1156.756500 1172.714000 1183.219300 1207.224000 1212.862500 1220.296400 1228.466200 1245.831700 1255.148400 1267.887200 1269.348800 1295.470600 1311.667800 1315.785200 1323.254200 1332.526100 1347.308900 1354.998700 1369.955700 1370.532500 1384.044500 1384.546500 1397.912400 1404.414200 1415.676400 1432.786500 1436.112800 1447.828200 1465.639000 1475.338700 1477.273300 1481.666400 1482.359900 1494.708900 1500.007500 1507.362600 1510.375200 1516.886200 1549.894100 1565.524100 1609.014100 1619.366900 1626.377400 1635.888300 1636.520600 1653.297300 1714.011400 1714.628700 1749.417000 1754.097800 1802.052900 3062.753400 3077.146600 3078.089700 3116.658000 3128.627400 3132.438900 3143.507600 3175.802700 3177.938300 3202.879500 3213.288500 3213.513500 3219.053600 3219.487200 3226.804800 3227.102000 3238.321700 3238.409900

3-10. Geometrical parameters of PDI-S.

Center Number	Atomic Number	Atomic Type	Coordinates (Angstroms) X	Y	Z
1	6	0	3.251085	−1.536120	−0.543580
2	6	0	3.352811	0.941920	−0.727677
3	7	0	3.950668	−0.330806	−0.698585
4	6	0	−0.355293	−2.554568	−0.139486
5	6	0	−1.048551	−1.341360	−0.151798
6	6	0	−0.303627	−0.128107	−0.302391
7	6	0	1.119328	−0.195398	−0.435253
8	6	0	1.777306	−1.450309	−0.414617
9	6	0	1.037376	−2.612381	−0.267643
10	6	0	−0.946664	1.150883	−0.324119
11	6	0	1.877598	0.991979	−0.586727
12	1	0	1.556634	-3.564578	−0.253091
13	6	0	1.235057	2.219430	−0.605536
14	6	0	−0.156748	2.293476	−0.475791
15	1	0	1.829894	3.119000	−0.721564
16	8	0	4.032786	1.950924	−0.866360
17	8	0	3.852843	−2.605073	−0.517070
18	6	0	−4.693919	−2.340302	0.267913
19	6	0	−5.336500	−1.112986	0.251183
20	6	0	−4.577427	0.074034	0.099940
21	6	0	−3.154769	0.006769	−0.034805
22	6	0	−2.511978	−1.272139	−0.014452
23	6	0	−3.302078	−2.414571	0.136645

24	1	0	–5.288543	–3.239849	0.384665
25	6	0	–5.235219	1.328774	0.082984
26	6	0	–2.410066	1.220044	–0.186019
27	6	0	–3.103530	2.433036	–0.196852
28	6	0	–4.496145	2.490914	–0.064514
29	1	0	–5.015581	3.443095	–0.075369
30	6	0	–6.708744	1.411745	0.221958
31	6	0	–6.812940	–1.063805	0.393664
32	7	0	–7.407392	0.207172	0.368864
33	8	0	–7.314464	2.478015	0.211998
34	8	0	–7.488120	–2.078402	0.527014
35	1	0	–2.845498	–3.396394	0.156167
36	1	0	–0.613566	3.275139	–0.496120
37	1	0	–2.568085	3.367516	–0.309511
38	1	0	–0.890993	-3.488910	–0.027200
39	6	0	–8.867065	0.309414	0.505953
40	6	0	5.417542	–0.412558	–0.842206
41	1	0	–9.284521	0.806561	–0.372109
42	1	0	5.635035	–1.288765	–1.453913
43	1	0	–9.264018	–0.698078	0.601699
44	1	0	–9.112938	0.905129	1.387654
45	1	0	5.740970	0.491136	–1.356464
46	6	0	6.104805	–0.526045	0.525396
47	1	0	5.949601	0.377159	1.122447
48	1	0	5.738375	–1.399694	1.067807
49	6	0	8.505480	0.862864	0.981797
50	8	0	7.772525	1.714009	1.436759
51	6	0	10.008005	1.023146	0.883446

52	1	0	10.517184	0.083987	0.655869
53	1	0	10.225026	1.746418	0.089838
54	1	0	10.380727	1.433757	1.825719
55	16	0	7.917002	−0.723103	0.344895

3-11. Vibrational frequencies of PDI-S.

11.594400 16.988000 22.212800 25.354300 38.290400 44.206900 58.733600 64.340300 72.707900 79.308100 83.892900 94.629500 118.076000 122.019200 127.938400 161.662300 176.589200 188.886100 200.072800 229.107600 238.891800 247.104100 268.757500 272.497300 314.261000 323.067300 331.520800 362.028800 365.166200 380.930700 383.621500 393.243100 397.324900 412.002300 434.385300 442.465300 446.209400 458.509000 468.143000 477.940100 481.661700 492.827900 517.806700 530.217100 532.522500 544.035500 575.166800 582.743900 598.130500 611.843500 618.233800 623.927000 624.397600 648.365500 657.317500 686.954000 704.902000 716.295800 724.269900 729.855700 747.523200 753.081000 757.844700 758.643600 765.922000 770.913800 803.433400 806.625100 832.046300 852.975900 862.850900 865.128400 865.715200 885.893700 921.043800 956.377500 976.837200 980.744600 988.225000 990.641300 995.638700 998.669500 1017.011400 1027.247200 1029.996000 1045.470100 1057.713600 1080.170200 1104.234500 1127.104100 1138.252200 1152.391200 1156.484000 1177.111000 1189.162900 1210.587500 1215.762100 1225.888500 1243.852400 1253.921000 1266.892500 1281.355400 1294.676800 1310.183100 1314.568100 1322.018100 1331.091300 1347.394400 1364.363200 1377.892200 1380.082700 1385.966400 1390.880600 1393.400800 1409.307600 1418.767300 1432.386400 1446.743100 1464.882100 1470.842500 1472.494500 1476.073800 1481.152400 1481.551900 1494.702800 1499.845600 1507.195000 1509.872900 1549.926400 1565.505900 1608.927900 1619.124400 1626.287200 1635.349700 1636.748000 1652.661500 1713.360900 1713.677800 1749.212400 1753.710600 1776.166800 3054.070200 3076.719600 3081.530100 3114.348800 3128.891000 3142.907400 3147.373400 3152.080900 3180.184600 3202.484900 3211.996800 3212.126200 3219.078500 3219.310200 3226.212600 3226.411100 3237.373900 3237.457400

3-12. Geometrical parameters of PDI-Se.

Center Number	Atomic Number	Atomic Type	Coordinates (Angstroms) X	Y	Z
1	6	0	2.764259	−1.446584	−0.678237
2	6	0	2.822429	1.026003	−0.931075
3	7	0	3.442204	−0.235976	−0.872181
4	6	0	−0.808538	−2.508120	−0.124681
5	6	0	−1.523517	−1.308441	−0.169097
6	6	0	−0.803591	−0.088385	−0.374686
7	6	0	0.615494	−0.137551	−0.548085
8	6	0	1.294216	−1.380528	−0.508773
9	6	0	0.580258	−2.548136	−0.294043
10	6	0	−1.467188	1.179615	−0.408746
11	6	0	1.349682	1.055847	−0.758292
12	1	0	1.116926	−3.490167	−0.261193
13	6	0	0.686564	2.271873	−0.795559
14	6	0	−0.701525	2.328644	−0.622905
15	1	0	1.262662	3.176572	−0.957054
16	8	0	3.478957	2.042699	−1.117815
17	8	0	3.380865	−2.507483	−0.651934
18	6	0	−5.148698	−2.360724	0.288765
19	6	0	−5.807883	−1.142137	0.290524
20	6	0	−5.068515	0.055662	0.127310
21	6	0	−3.648156	0.008179	−0.036151
22	6	0	−2.986344	−1.260965	−0.019400
23	6	0	−3.758366	−2.415352	0.134013

24	1	0	–5.729163	–3.268850	0.410247
25	6	0	–5.743661	1.301246	0.125412
26	6	0	–2.925237	1.230596	–0.216724
27	6	0	–3.634867	2.434193	–0.209167
28	6	0	–5.024035	2.473368	–0.038761
29	1	0	–5.556292	3.418507	–0.035179
30	6	0	–7.214438	1.363757	0.298999
31	6	0	–7.280810	–1.113357	0.467391
32	7	0	-7.892980	0.149405	0.459117
33	8	0	-7.834875	2.421603	0.305740
34	8	0	–7.938949	–2.137349	0.613987
35	1	0	–3.288211	–3.390936	0.137228
36	1	0	–1.174915	3.301947	–0.659845
37	1	0	–3.115003	3.375582	–0.336640
38	1	0	–1.324318	–3.445567	0.042110
39	6	0	-9.350365	0.230941	0.629720
40	6	0	4.913568	–0.298509	–1.001189
41	1	0	–9.794807	0.723246	–0.237784
42	1	0	5.150725	-1.093554	–1.710857
43	1	0	–9.730737	–0.782215	0.732796
44	1	0	–9.584414	0.821956	1.517772
45	1	0	5.235904	0.660643	–1.401993
46	6	0	5.556447	–0.574200	0.357943
47	1	0	5.320825	0.218531	1.074213
48	1	0	5.229090	-1.535443	0.753959
49	6	0	7.826174	1.099158	1.167149
50	8	0	6.931594	1.771994	1.622824
51	6	0	9.283161	1.500525	1.185528

52	1	0	9.464840	2.176817	2.024125
53	1	0	9.941420	0.629807	1.246946
54	1	0	9.505994	2.028947	0.251253
55	34	0	7.508972	−0.632684	0.296936

3-13. Vibrational frequencies of PDI-Se.

13.879600 17.835700 25.294200 30.772300 41.659500 55.792500 63.277600 70.025300 75.100400 79.032900 86.393400 95.854100 113.540800 123.771000 129.239200 160.904300 168.092400 186.674300 202.835600 216.169800 236.830000 237.181800 270.255100 273.577000 307.037600 315.584700 322.173100 338.070900 360.123100 371.891300 379.239600 386.039700 393.279900 398.636700 412.013700 434.058100 442.107200 444.911800 447.121300 466.332900 477.767300 479.633800 506.268700 517.717800 527.620100 541.327400 575.203200 576.861600 580.889700 596.719900 607.115900 618.493300 623.962600 646.607500 658.252000 681.474200 701.849200 707.614600 718.255600 725.468500 734.039800 739.183900 757.710800 759.505400 763.988800 769.045300 801.868700 806.500700 831.922800 852.957200 862.700700 865.313800 865.775300 884.574500 920.290700 951.318200 962.444500 975.389400 987.235000 990.373800 995.445900 998.314300 1014.968100 1020.283800 1029.649300 1051.714500 1057.508700 1080.146100 1104.132300 1120.458600 1137.241700 1152.622400 1156.318300 1177.312400 1189.600200 1210.713800 1215.677500 1225.329600 1243.329900 1254.372400 1266.997600 1271.618100 1302.604100 1310.086400 1314.700200 1322.488300 1331.150400 1347.715600 1361.146200 1373.770400 1380.219900 1384.363300 1389.873800 1391.250000 1408.740100 1418.605800 1432.695500 1446.858600 1463.890000 1465.421100 1470.154100 1478.532700 1478.802700 1483.478500 1494.775700 1499.817800 1507.283800 1510.114800 1549.896800 1565.555400 1609.147200 1619.258800 1626.353100 1635.656900 1636.843900 1653.018400 1712.726500 1713.467600 1748.966900 1753.496900 1788.626900 3049.268900 3076.631300 3083.665900 3105.977100 3122.962200 3142.770000 3153.404700 3159.244500 3182.875300 3202.450800 3212.051300 3212.612300 3219.116600 3219.334600 3226.220600 3226.641800 3237.242000 3237.610400

Table S3. The highest occupied molecular orbital (HOMO) and lowest unoccupied molecular orbital (LUMO) energies of PDI-Ph calculated by different methods together with 6-31+G(d,p) basis set.

**Methods and Basis Sets**	**HOMO**	**exp** [a]	**LUMO**	**exp** [a]
B3LYP	−6.28	−5.98	−3.80	−3.92
PBE1PBE	−6.46		−3.70	
MPW1B95	−6.30		−3.16	
PBE33	−6.50		−3.20	
PBE38	−6.64		−3.10	
MPWB1K	−6.74		−2.84	

[a] ref 28.

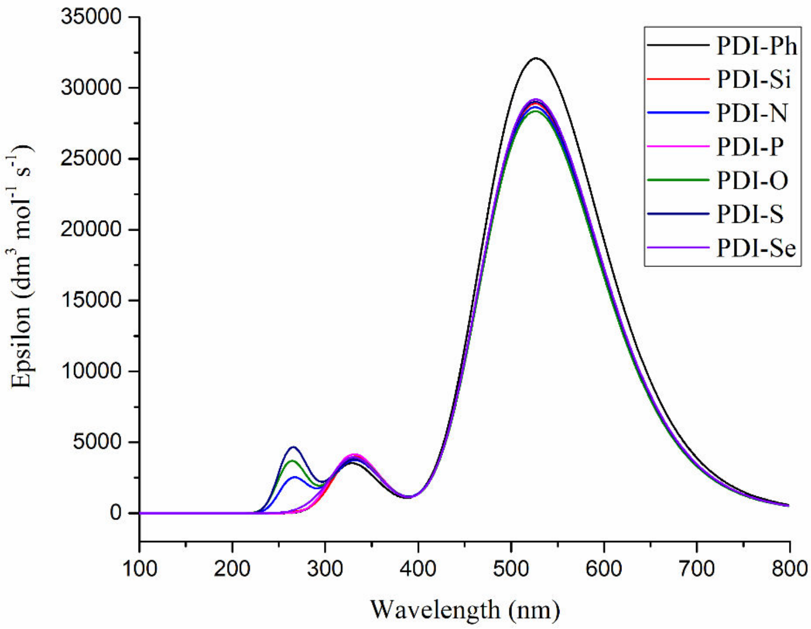

Figure S1. TD-DFT UV-visible absorption spectrum for investigated molecules.

Table S4. Absorption maximum wavelength (λ_{max}), oscillator strength, excitation energy, electronic transition and main configuration for the PDI derivatives.

**Molecules**	λ_{max}	λ_{max}, **exp** [a]	**Oscillator Strength**	**Excitation Energy**	**Electronic Transistion**	**Main Configu-ration**
PDI-Ph	527.02	550	0.79	2.35	$S_0 \rightarrow S_1$	H → L 0.71
PDI-Si	525.95		0.71	2.36	$S_0 \rightarrow S_1$	H → L 0.71
PDI-N	525.90		0.71	2.36	$S_0 \rightarrow S_1$	H → L 0.71
PDI-P	526.15		0.72	2.36	$S_0 \rightarrow S_1$	H → L 0.71
PDI-O	525.84		0.70	2.36	$S_0 \rightarrow S_1$	H → L 0.71
PDI-S	526.08		0.72	2.36	$S_0 \rightarrow S_1$	H → L 0.71
PDI-Se	526.36		0.72	2.36	$S_0 \rightarrow S_1$	H → L 0.71

[a] ref 28.

Table S5. The hole reorganization energy λ_{hole} (eV), centroid to centroid distance (d, Å), hole transfer integral V_{hole} (eV), hole transfer rate k_{hole} (s^{-1}) and hole mobility μ_{hole} ($cm^2V^{-1}s^{-1}$) for studied molecules.

Molecule	λ_{hole}	d	V_{hole}	K_{hole}	μ_{hole}
PDI-Ph	0.16	3.86	5.72×10^{-2}	2.90×10^{13}	0.84
PDI-Si	0.11	4.56	0.22	8.40×10^{14}	33.78
PDI-N	0.18	4.38	0.34	7.94×10^{14}	29.46
PDI-P	0.11	5.20	3.18×10^{-3}	1.76×10^{11}	9.20×10^{-3}
PDI-O	0.17	4.41	0.46	1.65×10^{15}	62.06
PDI-S	0.16	5.83	6.75×10^{-2}	4.03×10^{13}	2.65
PDI-Se	0.13	4.33	0.27	9.58×10^{14}	34.74

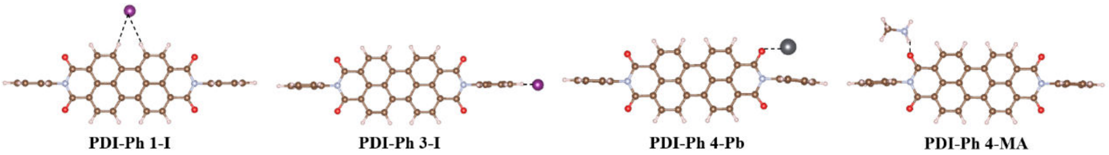

Figure S2. Interactions between H or O in PDI-Ph with I^-, Pb^{2+} or MA^+.

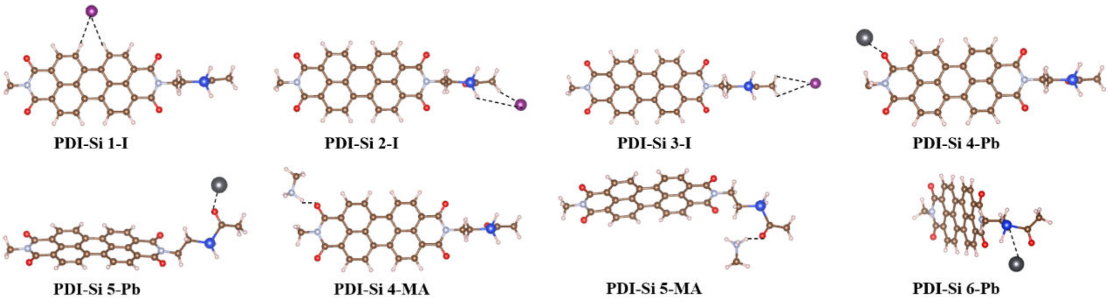

Figure S3. Interactions between H, O or Si in PDI-Si with I^-, Pb^{2+} or MA^+.

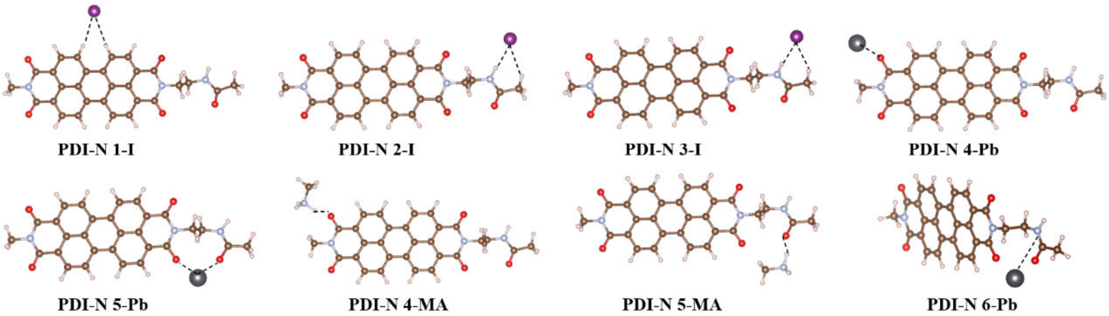

Figure S4. Interactions between H, O or N in PDI-N with I^-, Pb^{2+} or MA^+.

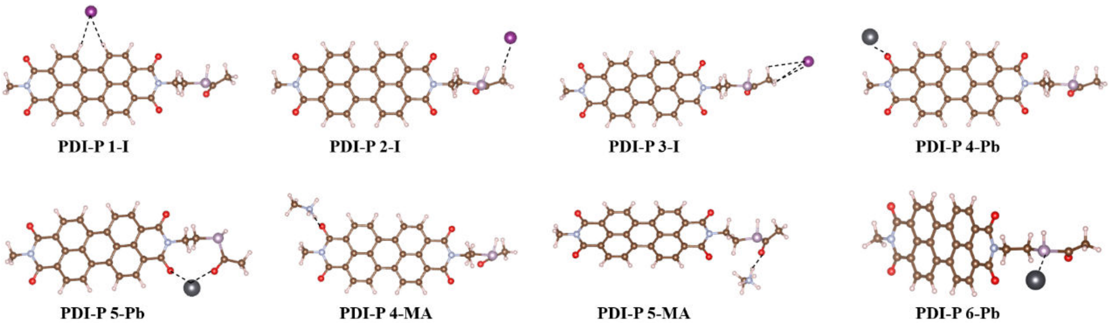

Figure S5. Interactions between H, O or P in PDI-P with I^-, Pb^{2+} or MA^+.

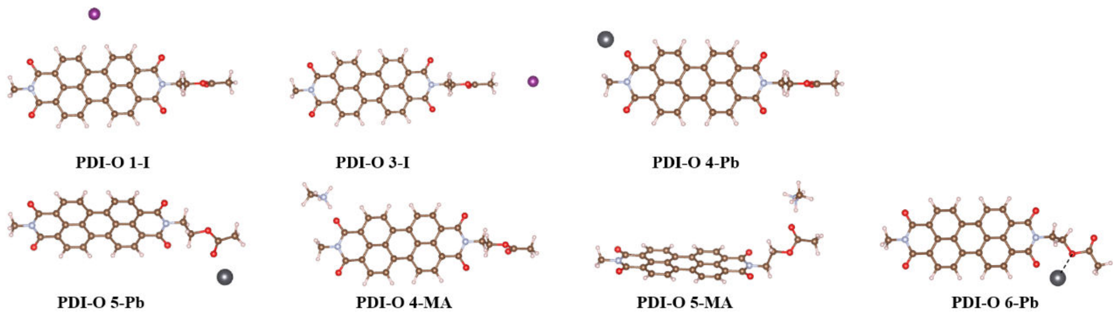

Figure S6. Interactions between H or O in PDI-O with I^-, Pb^{2+} or MA^+.

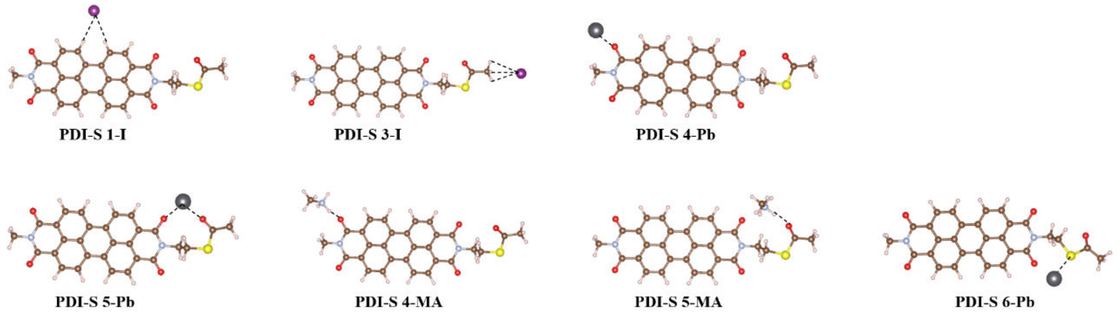

Figure S7. Interactions between H, O or S in PDI-S with I^-, Pb^{2+} or MA^+.

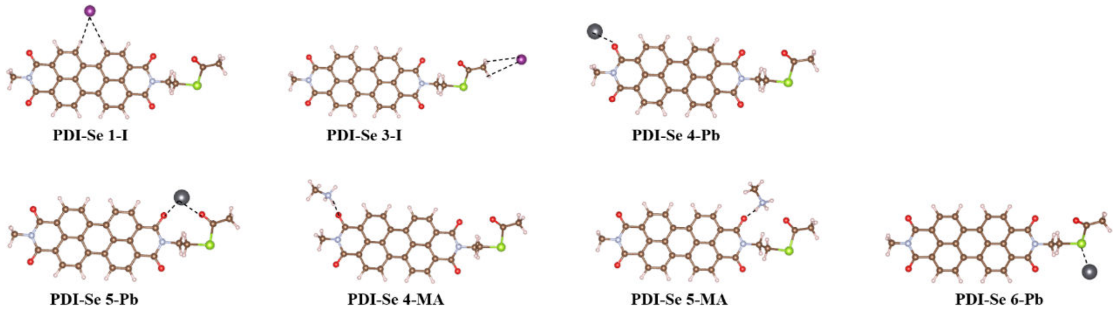

Figure S8. Interactions between H, O or Se in PDI-Se with I^-, Pb^{2+} or MA^+.
